# Clinical Outcome of Viral Respiratory Tract Infections in Hospitalized Adults in Norway: High Degree of Inflammation and Need of Emergency Care for Cases With Respiratory Syncytial Virus

**DOI:** 10.3389/fmed.2022.866494

**Published:** 2022-04-29

**Authors:** Sara Debes, Jon Birger Haug, Birgitte Freiesleben de Blasio, Jonas Christoffer Lindstrøm, Christine Monceyron Jonassen, Susanne Gjeruldsen Dudman

**Affiliations:** ^1^Center for Laboratory Medicine, Østfold Hospital Trust, Østfold, Norway; ^2^Institute of Clinical Medicine, Faculty of Medicine, University of Oslo, Oslo, Norway; ^3^Department of Infection Control, Østfold Hospital Trust, Østfold, Norway; ^4^Division of Infection Control and Environmental Health, Department of Methods Development and Analytics, Norwegian Institute of Public Health, Oslo, Norway; ^5^Department of Biostatistics, Institute of Basic Medical Sciences, Centre for Biostatistics and Epidemiology, University of Oslo, Oslo, Norway; ^6^Department of Virology, Norwegian Institute of Public Health, Oslo, Norway; ^7^Department of Microbiology, Oslo University Hospital, Oslo, Norway

**Keywords:** respiratory syncytial virus – RSV, viral respiratory tract infection, Adults, antibiotic, hospitalization, influenza, human metapneumovirus (hMPV)

## Abstract

**Background:**

The clinical features and outcomes of viral respiratory tract infections (RTIs) in adults have not been thoroughly studied, especially the respiratory syncytial virus (RSV) disease burden. It has become apparent that outbreaks of RSV in the elderly are associated with increased hospitalization rates. However, little data exists on the severity of such viral RTIs in adults, particularly the need for hospitalization, respiratory support and intensive care.

**Methods:**

We conducted a retrospective observational single-center study at Østfold Hospital Trust, Norway, during three winter seasons 2015–2018. Patients ≥18 years with either influenza A, influenza B, RSV A/B, human metapneumovirus, parainfluenza virus 1–4 or adenovirus detected in respiratory specimens were included, if they were hospitalized 14 days prior or following the detection date, with signs of RTI. Hospital records on treatment and outcome were investigated, as well as mortality of all causes up to 30 days from discharge.

**Results:**

Of the 1222 infection events that were included, influenza A was the most frequent virus detected (39%), while 179 infection events (14.6%) were due to RSV. Influenza B counted for 24% of the infection events, human metapneumovirus 13%, parainfluenza virus 9% and adenovirus 1%. Patients admitted with RSV more often suffered from COPD and congestive heart failure than patients with influenza A. In addition, RSV patients were overrepresented in the urgent response NEWS score (National Early Warning Score) category ≥5. RSV patients also showed signs of more severe inflammation, with WBC ≥11.1 × 10^9^/L and CRP >100 mg/L, and they were more often treated with antibiotic agents during their hospital stay. However, we found no differences in the need for ICU admission or mortality.

**Conclusion:**

Patients with RSV had more often high values for markers of inflammation and elevated NEWS score when compared to patients hospitalized with other common respiratory viruses. Taken into account that they suffered more frequently from comorbidities like COPD, these patients needed hospitalization more urgently. These findings highlight the need for further investigations on RSV disease in adults and the elderly.

## Introduction

While there is a large amount of published literature on viral respiratory tract infections (RTIs) in children, the clinical features and outcomes of such infections in adults have not been studied to the same degree (1, 2). Although extensive research has been carried out on influenza infections in adults, fewer studies have investigated other viral RTIs like respiratory syncytial virus (RSV) or human metapneumovirus (hMPV) regarding the extent of their disease burden. Falsey and her collaborators have some recent studies showing the impact of RSV and hMPV in adults (3–5), and Walsh has highlighted that respiratory syncytial virus is an illness of all ages (6). In contrast to influenza, there are no vaccines or antivirals available to prevent and treat RSV and hMPV. The incidence of severe pneumococcal pneumonia was reduced after the introduction of systematic vaccination both in children and adults, resulting in an increase in the percentage of viral RTIs (7). Recently, diagnostic advances with nucleic acid-based multiplex methods have shown that viral respiratory pathogens can be detected in a high percentage of adult cases in primary care and hospital settings (7). It has become apparent that outbreaks of RSV in the population are associated with increased rates of hospitalization in adults, but the extent of the problem needs to be investigated (2). Little is known about the severity of different viral RTIs in adults and the resulting mortality rates. To this date, less data exists on the health care burden of such viral RTIs in adults, the need for hospitalization, respiratory support and intensive care.

In a study from 2014, the hospitalization rate for RSV positive elderly patients with moderate to severe influenza-like illness was twice that of other respiratory viruses and five times the rate of those who tested positive for influenza A (3). In another review from 2019, RSV was found to be the third most commonly identified viral cause of hospital admissions (8). Immunity to RSV is incomplete, and reinfection is common despite relatively high levels of serum neutralizing antibodies in adults (6).

A recently published systematic review including 20 studies concluded that the disease burden of RSV acute respiratory infection among adults with comorbidities is substantial, with limited data available (9). RSV has also in studies been associated with greater odds of pneumonia, intensive care unit admission, exacerbation of COPD, and higher mortality, compared to influenza infection (10–13).

In this study, we investigated the clinical outcome of viral RTIs, the need for emergency care and 30-days mortality in hospitalized adults during winter seasons 2015–2018 in Norway.

## Materials and Methods

We conducted a retrospective observational study during a 3 years period at the Østfold Hospital Trust, a 500-bed hospital in south-eastern Norway, covering approximately 300,000 inhabitants.

Patients ≥18 years with at least one of the following viruses detected in respiratory specimens from week 40 to week 20, in 2015–2018 were included: influenza A, influenza B, RSVA + B, human metapneumovirus (hMPV), parainfluenza virus 1–4, adenovirus and enterovirus. The viral agent was detected with the multiplex polymerase chain reaction Seegene Allplex Respiratory Panel 1 and 2 assays (Seegene Inc., Seoul, South Korea) at Østfold Hospital Trust, Norway. Extraction of total nucleic acid was performed with NucliSENS Easymag (bioMérieux) in 2015–2016 and thereafter MagNA Pure 96 Instrument (Roche) according to the manufacturer’s instructions.

Testing of specimens with the Seegene panel was performed on all work days during the influenza season with sufficient capacity for the demand. Therefore there was a maximum waiting time of 24 h on weekdays increasing to 50 h during weekends for detecting a viral RTI.

From the hospital’s activity data register, we identified the hospitalized patients who were positive for at least one of the respiratory viruses, and retrieved information on their hospital stay, from 14 days before the virus detection date, until 14 days after. We then retrieved the hospital’s electronic patient journal (EPJ) on these patients. The EPJs were investigated, and the patient was included if there was information in the journal on symptoms of respiratory tract infection (RTI). Hospital stays of less than 5 h duration were excluded.

The hospital admission was defined as an infection event (IE), and all readmissions within a 14 days post-discharge period with an identical virus finding were included in the same IE. Readmissions within 14 days post-discharge, with a different virus finding, were categorized as a new infection event.

We registered antimicrobial therapy, including oseltamivir administration, during hospitalization and at discharge. In addition, we defined healthcare-associated infection (HAI) as symptoms of RTI that occurred more than 48 h from admission.

Background medical conditions were registered and standardized using the Charlson comorbidity score (14, 15). To assess the severity of eventual pneumonia and the need for hospitalization, we used the scoring tool CRB-65 (16). When assessing a patient with suspected pneumonia, the recommendation is to score with CRB65 to evaluate whether the patient is better suited for home care, hospitalization, or in need of urgent hospital treatment (17). However, none of the included patients had been scored with CRB65 at admission; instead, we made this assessment retrospectively based on clinical data from the EPJ.

NEWS score (National Early Warning Score) is a tool to quickly detect changes in the patient’s vital signs and clinical condition to prevent deterioration. We evaluated the disease severity by reviewing the NEWS value at admission and the maximum NEWS value during the hospital stay. We also examined the need for admission to the level 2 or level 3 Intensive care units and the need for respiratory support.

Biochemical markers, including C-reactive protein values and leukocyte count, were recorded both at admission and the maximum value during the IE, as well as chest X-ray and CT findings.

In-hospital mortality and 30 days mortality post-discharge of all causes from the Norwegian Patient Registry were counted.

Primary outcomes were in-hospital and 30 days all-cause mortality, admission to Intensive Care Unit (ICU) and need for respiratory support.

### Statistical Analysis

For statistical analyses, we used SPSS (IBM SPSS Statistics for Windows, version 25.0. Armonk, NY: IBM Corp).

Categorical variables are presented as frequencies and percentages, while continuous variables are described as mean, median, standard deviation and interquartile range (IQR).

Statistical analysis was performed by using Chi-square test, One way ANOVA, Independent-samples median test, logistic and linear regression. We adjusted for patient age and gender in all regression analyses.

*P*-values less than 0.05 were considered statistically significant.

## Results

### Patient Characteristics at Hospital Admission

#### Demographic Data

**FIGURE 1:**
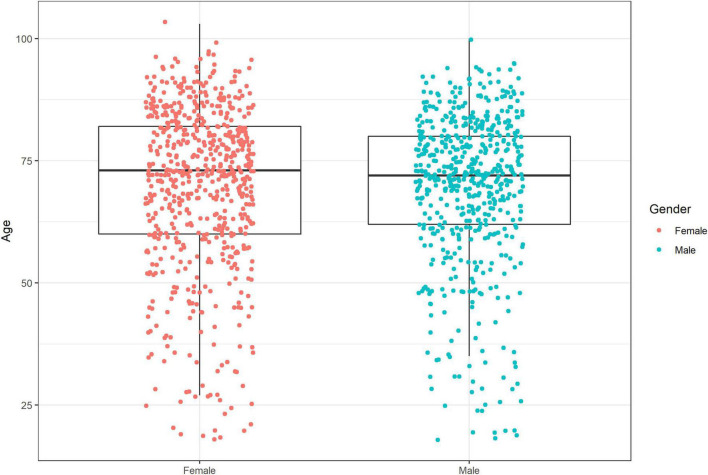
Boxplot of age and gender of hospitalized adults with confirmed viral RTI, in three consecutive seasons 2015–2018.

We included 1222 infection events (IEs) requiring hospital treatment in 1182 adults over three consecutive winter seasons. The mean age was 69.6 years (SD 16.1), the median age was 72 (range 18–103), and 53% of the patients were female (Figure 1 and Table 1). The majority (91%) of the patients were admitted from home; only 5% were from short-term and 4% from long-term care facilities. When investigating the population admitted from long-term care facilities, we found no difference in the distribution of the different virus types (*p* = 0.814, Chi-square). Five per cent of the patients had received chemotherapy, and 16% had been treated with prednisone within 4 and 2 weeks prior to admission, respectively.

**TABLE 1:** Baseline patient characteristics and initial findings in hospitalized adults with confirmed viral RTI, in three consecutive seasons 2015–2018.

	All Infection events, IE (*n* = 1222)	Adenovirus (*n* = 10, 0.8%)	Parainfluenza 1–4 (*n* = 111, 9.1%)	Influenza A (*n* = 473, 38.7%)	Influenza B (*n* = 294, 24.1%)	hMPV (*n* = 155, 12.7%)	RSVAB (*n* = 179, 14.6%)	*P*-value
**Demographic data**								
Gender female (%)	648 (53)	8 (80)	57 (51.4)	244 (51.6)	148 (50.3)	85 (54.8)	106 (59.2)	0.366*
Age Mean (SD)	69.6 (16.1)	39.5 (12.5)	68.7 (17.3)	69.5 (15.8)	69.3 (17.1)	70.0 (15.5)	72 (13.6)	0.340**
**Comorbidity**								
Charlson Score mean (SD)	4.7 (2.6)	2.3 (2.9)	5.0 (2.7)	4.5 (2.4)	4.7 (2.8)	4.8 (2.5)	4.9 (2.5)	0.126**
COPD (%)	447 (36.6)	1 (10)	48 (43.2)	162 (34.2)	89 (30.3)	65 (41.9)	82 (45.8)	0.002*
Prior myocardial infarction (%)	186 (15.2)	0	16 (14.4)	66 (14.0)	42 (14.3)	27 (17.4)	35 (19.6)	0.408*
Congestive heart failure (%)	169 (13.8)	0	22 (19.8)	55 (11.6)	34 (11.6)	26 (16.8)	32 (17.9)	0.040*
Solid malignant tumor (%)	122 (10)	2 (20)	17 (15.3)	32 (6.8)	37 (12.6)	13 (8.4)	21 (11.7)	0.015 *
Hematopoietic malignancy (%)	24 (2.0)	0	3 (2.7)	11 (2.3)	2 (0.7)	3 (2.0)	5 (2.8)	0.439*
Organ TX (%)	32 (2.6)	0	5 (4.5)	8 (1.7)	8 (2.7)	7 (4.5)	4 (2.2)	0.249*
Cerebrovascular disease (%)	176 (14.4)	0	20 (18.0)	64 (13.5)	43 (14.6)	22 (14.2)	27 (15.1)	0.821*
Renal failure(%)	121 (9.9)	0	11 (9.9)	36 (7.6)	36 (12.2)	23 (14.8)	15 (8.4)	0.056*
Liver failure (%)	20 (1.6)	0	2 (1.8)	6 (1.3)	6 (2.0)	4 (2.6)	2 (1.1)	0.762*
Diabetes (%)	233 (19.1)	1 (10)	25 (22.5)	94 (19.9)	51 (17.3)	31 (20.0)	31 (17.3)	0.726*
**Findings at admission:**								
Duration of symptoms in days, median (IQR)	4.0 (5.0) nd = 62	3.0 (4.0)	4.0 (5.0) nd = 2	3.0 (5.0) nd = 21	4.0 (5.0) nd = 22	4.0 (4.0) nd = 8	4.0 (5.0) nd = 9	0.08***
Temperature ≥38C° (%)	632 (52.1) nd = 8	4 (40)	56 (50.5)	255 (54.6) nd = 6	152 (52.1) nd = 2	83 (53.5)	82 (45.8)	0.372*
Heart rate >100 bpm (%)	396 (32.4)	6 (60)	40 (36)	155 (32.8)	78 (26.5)	54 (34.8)	63 (35.2)	0.173*
Respiratory rate >20 per minute (%)	621 (50.8)	5 (50)	60 (54.1)	233 (49.3)	157 (53.4)	77 (49.7)	89 (49.7)	0.758*
O2 Saturation <90% (%)	232 (19%) nd = 1	1 (10)	16 (14.1)	85 (18.0)	58 (19.8) nd = 1	29 (18.7)	43 (24.0)	0.299*
Arterial blood gas test (%):	711 (58.2)	2 (20)	71 (64)	275 (58.1)	141 (48)	95 (61.3)	127 (70.9)	<0.001*
**CRP mg/L**								
Mean (SD)	89.6 (91.8) nd = 1	132.4 (76.2)	80.9 (87.8)	90.5 (88.4) nd = 1	81.2 (91.0)	92.5 (99.8)	101.7 (96.5)	0.150**
CRP ≥ 100 (%)	388 (31.8)	7 (70)	30 (27)	150 (31.8)	87 (29.6)	40 (25.8)	74 (41.3)	0.018*
**WBC10** ^**9**^ **/L**								
Mean (SD)	9.3 (4.4) nd = 3	9.2 (5.9)	9.8 (4.2)	9.1 (4.0) nd = 1	8.3 (4.6) nd = 1	9.6 (4.6)	10.5 (4.3) nd = 1	<0.001**
WBC ≥11.1 (%)	321 (26.3)	4 (40)	34 (30.6)	119 (25.2)	56 (19.1)	40 (25.8)	68 (38.2)	<0.001*
**CRB 65**								
Likely suitable for home treatment (%)	304 (24.9)	10 (100%)	33 (29.7)	116 (24.5)	75 (25.5)	35 (22.6)	35 (19.6)	0.205*
Consider hospital referral (%)	866 (70.9)	0	74 (66.7)	335 (70.8)	201 (68.4)	116 (74.8)	140 (78.2)	0.205*
Urgent hospital admission (%)	52 (4.3)	0	4 (3.6)	22 (4.7)	18 (6.1)	4 (2.6)	4 (2.2)	0.205*
**NEWS**								
Mean (SD)	4.0 (2.7) nd = 107	3.4 (2.6)	4.4 (2.6) nd = 2	4.0 (2.7) nd = 54	3.6 (2.8) nd = 14	4.3 (2.6) nd = 14	4.6 (2.8) nd = 16	<0.001**
≥5 points	452 (40.5)	4 (40)	48 (47.1)	152 (36.3)	96 (34.3)	66 (46.8)	86 (52.8)	<0.001*

*P-value for categorical variables: Chi-square.

**P-value for continuous variables: One-way ANOVA.

***P-value for median: Independent-samples median test.

*Adenovirus excluded from calculations.*

*IE = Infection event; Nd = No data; SD = Standard deviation; WBC = White blood cell count; CRP = C-reactive protein; Bpm = Beats per minute.*

*P-values less than 0.05 were considered statistically significant and shown in bold values.*

#### Comorbidities

We scored the patients with Charlson Comorbidity Index (14, 15) and found a mean age-adjusted score of 4.7 (SD2.6), with no significant difference when comparing the different respiratory viruses (*p* = 0.126, Chi-square). However, within specific variables in the Charlson scores, we found a higher number of RSV patients with chronic obstructive pulmonary disease (COPD) compared to influenza A OR 1.6 (95% CI 1.1–2.2, *p* = 0.013, logistic regression).

We found significantly fewer patients with solid malignant tumor in the influenza A group, only 6.8% of the patients in comparison with 10% when looking at all the virus types together OR 0.5 (95% CI 0.34–0.80, *p* = 0.003, logistic regression). On the contrary, compared to influenza A, the parainfluenza virus patient group had a significantly larger number of patients with solid malignant tumor, with OR 2.5 (95% CI 1.34–4.77, logistic regression).

Respiratory syncytial virus and parainfluenza positive patients were more often suffering from congestive heart failure than patients with influenza A, with 18% vs. 12%, OR 1.69 (95% CI 1.03–2.75, *p* = 0.037, logistic regression) and 20% vs. 12%, respectively, with OR 1.97 (95% CI 1.12–3.46, *p* = 0.019, logistic regression).

We found a higher number of patients suffering from renal failure in both the influenza B patient group and hMPV patient group, compared to influenza A, with OR 1.7 (95%CI 1.04–2.77, *p* = 0.035) and OR 2.16 (95%CI 1.23–3.8, *p* = 0.008), respectively.

Organ transplanted patients was admitted more often due to hMPV than influenza A, OR 2.91 (95%CI 1.02–8.31, *p* = 0.045).

#### Symptoms and Signs

On average, the patients reported developing new symptoms compatible with acute respiratory tract infection before admission for a median of 4 days (IQR 5). We found no significant difference between the virus types pertaining to the duration of symptoms, ranging up to 35 days (*p* = 0.08, Independent-samples median test). Twelve patients (1%) developed symptoms >48 h after admission and were identified as having a hospital-acquired infection (HAI).

Over half of the patients (53%) presented with fever (≥ 38° C) on admission, but with no significant difference between the virus types (*p* = 0.372, Chi-square). Also on admission, 51% of the patients were tachypneic, with a respiratory rate >20, but only the minority of all patients (32%) had tachycardia, defined as a heart rate >100.

We found no difference between the different viral RTIs concerning low oxygen saturation at a threshold of <90%, with 19% of all patients presenting with this condition (*p* = 0.299, Chi-square).

As for the clinical chemical tests at admission, RSV patients were overrepresented in the group with leukocytosis (WBC ≥11.1 × 10^9^/L), with OR 1.8 (95% CI 1.3–2.6, *p* = 0.001, logistic regression), when compared to influenza A (Table 2).

**TABLE 2:** Regression analyses of biochemical parameters, treatment variables and outcomes of hospitalized adults with the different viruses compared to influenza A, adjusted for age and gender.

	WBC ≥11.1	NEWS >5	Antibiotic treatment	CRP ≥100	Need for respiratory support	Need for ICU stay	Length of stay*	In-hospital Mortality	30 days mortality	Total mortality
**RSV**										
OR (95%CI)	1.82 (1.26–2.64)	1.90 (1.31–2.75)	2.25 (1.28–3.96)	1.54 (1.08–2.19)	1.02 (0.63–1.66)	1.078 (0.67–1.73)	*B* = 0.358 (−0.44–1.16)	1.05 (0.39–2.78)	0.825 (0.26–2.63)	1.124 (0.52–2.41)
*P*-value	**0.001**	**0.001**	**0.005**	**0.019**	0.937	0.757	0.380	0.929	0.746	0.765
**Human metapneumovirus**										
OR (95%CI)	1,029 (0.68–1.56)	1.529 (1.04–2.26)	1.538 (0.91–2.60)	0.747 (0.50–1.13)	0.681 (0.38–1.22)	0.699 (0.40–1.23)	*B* = −0.295 (−1.14–0.55)	0.820 (0.31–2.18)	0.786 (0.22–2.86)	0.990 (0.45–2.19)
*P*-value	0.894	**0.033**	0.109	0.162	0.194	0.214	0.491	0.691	0.715	0.980
**Influenza B**										
OR (95%CI)	0.702 (0.49–1.00)	0.926 (0.67–1.28)	0.954 (0.66–1.39)	0.901 (0.66–1.24)	0.539 (0.33–0.98)	0.681 (0.43–1.07)	*B* = −0.331 (−1.00–0.34)	0.802 (0.37–1.76)	1.061 (0.42–2.66)	0.862 (0.47–1.59)
*P*-value	0.052	0.639	0.806	0.520	**0.014**	0.095	0.336	0.583	0.900	0.636
**Parainfluenza 1–4**										
OR (95%CI)	1.309 (0.83–2.06)	1.600 (1.03–2.49)	1.202 (0.69–2.10)	0.789 (0.50–1.25)	1.180 (0.67–2.09)	0.901 (0.49–1.64)	*B* = 0.174 (−0.78–1.13)	0.694 (0.24–0.1.98)	1.130 (0.31–4.14)	0.777 (0.34–1.80)
*P*-value	0.245	**0.037**	0.519	0.316	0.569	0.734	0.721	0.494	0.854	0.554

*Analyzed with linear regression.

*CI = Confidence interval; Sig = Significance; WBC = White blood cell count; CRP = C-reactive protein.*

*P-values less than 0.05 were considered statistically significant and shown in bold values.*

The CRP in patients with RSV were more frequently detected at levels ≥100 mg/L, with OR 1.5 (95% CI 1.1–2.2, *p* = 0.019, logistic regression) when compared to influenza A.

### Severity Scoring

#### CRB65

The vast majority (71%) belonged to category 2, i.e., patients to be considered for hospital referral. There were no significant differences in CRB65 scores between the different viruses (*p* = 0.205, Chi-square).

#### NEWS Score

We found that RSV patients were overrepresented when compared to influenza A in the group with NEWS score at ≥5, which is the value for an urgent response, with OR 1.9 (95% CI 1.3–2.7, *p* = 0.001, logistic regression). This was also the case for hMPV, with OR 1.5 (95% CI 1.0–2.3, *p* = 0.033, logistic regression) and parainfluenza 1–4, with OR 1.6 (95% CI 1.0–2.5, *p* = 0.037, logistic regression).

### Case Investigation/Treatment

#### Virus Detection

**FIGURE 2:**
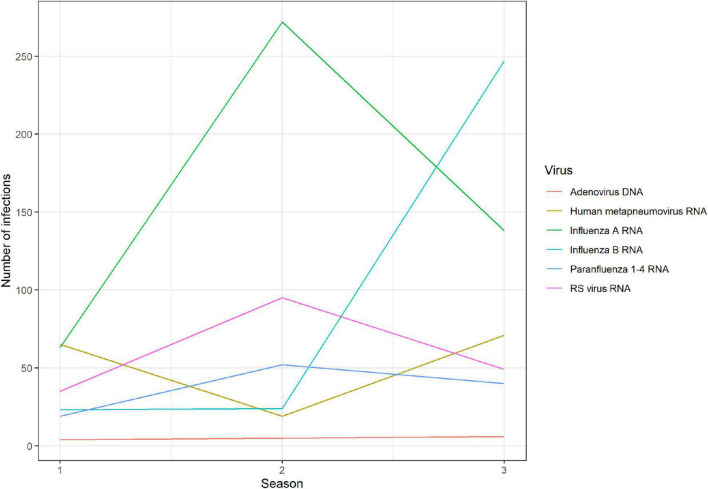
Number of admissions due to the different viral agents during the three winter seasons 2015–2018.

Overall, influenza A was the most frequent virus detected, accounting for 38.7%, followed by influenza B with 24.1%, whereas RSV and hMPV accounted for 14.6 and 12.7% of the IEs, respectively (Figure 2).

In the first season (2015/2016), hMPV was the most frequent virus detected (31%) followed by influenza A H1 (27%). The next season, (2016/2017), influenza virus A H3 was the most frequent (59%), while RSV counted for 21% of the infection events with RSV subtype B as the dominant subtype. The last season, (2017/2018) was dominated by influenza virus B (45%) and this season accounted for the highest percentage of the infection events when looking at the three seasons (45%) constituting the study period (18).

#### Radiological Findings

Almost all of the 1,182 patients (98.7%) underwent a chest X-ray, typical findings compatible with pneumonia were observed in 35.7% of the cases. We found no significant difference in signs of pneumonia when comparing the different viruses: rates varied from 32.6% for patients positive for influenza B to 41.7% for patients with RSV (*p* = 0.083, Chi-square). Only 252 patients were examined by chest computer tomography (CT), with typical findings of pneumonia found in 135 cases, showing no difference between virus types (*p* = 0.950, Chi-square) (Table 3).

**TABLE 3:** Case investigation and treatment during hospital stay in adults with viral RTI, 2015–2018.

	All Infection events, IE (*n* = 1222)	Adenovirus (*n* = 10, 0.8%)	Parainfluenza 1–4 (*n* = 111, 9.1%)	Influenza A (*n* = 473, 38.7%)	Influenza B (*n* = 294, 24.1%)	hMPV (*n* = 155, 12.7%)	RSVAB (*n* = 179, 14.6%)	*P*-value*
**Radiological investigation**								
Typical Chest X-ray findings compatible with pneumonia (%)	nd = 55		nd = 7	nd = 23	nd = 12	nd = 2	nd = 11	
Yes	417 (35.7)	5 (50)	34 (32.7)	147 (32.7)	92 (32.6)	69 (45.1)	70 (41.7)	0.083
Possible	48 (4.1)	0	4 (3.8)	21 (4.7)	13 (4.6)	3 (2.0)	7 (4.2)	0.083
**Hospital treatment**								
Oxygen therapy (%):	724 (59.2)	5 (50)	72 (64.9)	281 (59.4)	153 (52)	96 (61.9)	117 (65.4)	**0.026**
Admitted to ICU (%)	162 (13.3)	0	15 (13.5)	70 (14.8)	31 (10.5)	17 (11)	29 (16.2)	0.300
Non-invasive ventilation (CPAP, BiPAP) (%)	137 (11.2)	0	17 (15.3)	57 (12.1)	22 (7.5)	16 (10.3)	25 (14.0)	0.102
Respirator (%)	15 (1.2)	0	1 (0.9)	10 (2.1)	2 (0.7)	0	2 (1.1)	0.216
Oseltamivir treatment (%)	170 (13.9)	0	4 (3.6)	108 (22.8)	38 (12.9)	8 (5.2)	12 (6.7)	**<0.001**
Antibiotic treatment (%)	1021 (83.6)	8 (80)	93 (83.8)	385 (81.4)	237 (80.6)	135 (87.1)	163 (91.1)	**0.016**

*P-value for categorical variables: Chi-square. Adenovirus excluded from calculations.

*IE = Infection event; Nd = No data; SD = Standard deviation.*

*P-values less than 0.05 were considered statistically significant and shown in bold values.*

#### Antimicrobial Therapy

In total, 83% of the study population admitted with respiratory tract infection received antibiotics during the hospital stay or at discharge. Of the 244 patients (20%) treated prior to hospitalization, only 23 (9.4%) were not prescribed antibiotics during their hospital stay or at discharge.

Although not statistically significant, influenza B positive patients had a slightly lower treatment rate at 80.6%, with OR 0.95 (95% CI 0.7–1.4, *p* = 0.806, logistic regression). In the RSV group, 91% of the patients received antibiotics, a significantly higher rate than patients with influenza A, with OR 2.2 (95% CI 1.3–4, *p* = 0.007, logistic regression).

Only 13.9% of patients received antiviral treatment during the hospital stay, 23% in the influenza A group and only 13% in the influenza B group. Compared to influenza A, oseltamivir treatment in the group with influenza B was less common, with OR 0.5 (95% CI 0.3–0.8, *p* = 0.014, logistic regression).

#### Need for Respiratory Support (CPAP, BiPAP, and Respirator)

During their hospital stay, 12.4% of the patients needed respiratory support. Compared to influenza A, influenza B patients had less need for respiratory support, with OR 0.5 (95% CI 0.3–0.98, *p* = 0.014, logistic regression). We found no significant difference between RSV and influenza A patients.

#### Intensive Care Unit

A total of 13% of the patients required ICU care, with no difference when comparing the virus types (*p* = 0.300, Chi-square).

### Clinical Outcome

#### Length of Hospitalization and Discharge Status

**FIGURE 3:**
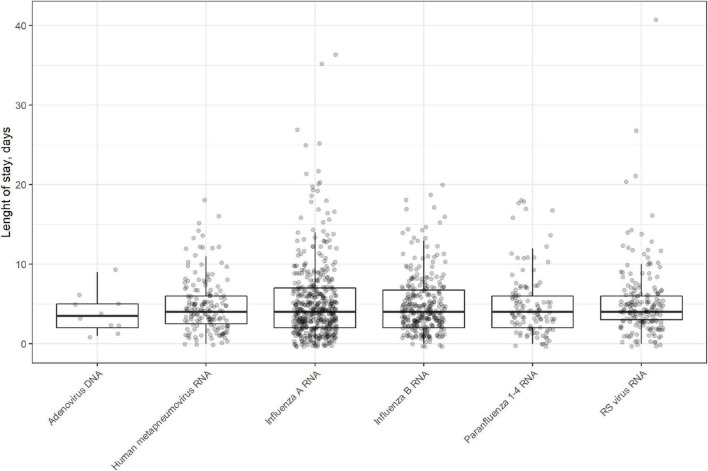
Boxplot of length of stay in days for the different virus types in patients hospitalized with viral RTI in 2015–2018.

The median length of hospital stay was 4 days (IQR 4). We found no significant difference in length of stay according to virus type (*p* = 0.912, Independent-samples median test) (Figure 3 and Table 4).

**TABLE 4:** Discharge status and clinical outcome for hospitalized adults with viral RTI during three consecutive winter seasons, 2015–2018.

	All Infection events, IE (*n* = 1222)	Adenovirus (*n* = 10, 0.8%)	Parainfluenza 1–4 (*n* = 111, 9.1%)	Influenza A (*n* = 473, 38.7%)	Influenza B (*n* = 294, 24.1%)	hMPV (*n* = 155, 12.7%)	RSVAB (*n* = 179, 14.6%)	*P*-value
Length of stay, days: Median (IQR)	4.0 (4.0)	3.5 (4.0)	4.0 (4.0)	4.0 (5.0)	4.0 (4.0)	4.0 (4.0)	4.0 (4.0)	0.912**
**Discharged to (%)**								
Home	844 (69.1)	9 (90)	81 (73)	323 (68.3)	194 (66)	112 (72.3)	125 (69.8)	0.794*
Short term care facility	285 (23.3)	0	21 (18.9)	112 (23.7)	80 (27.2)	33 (21.3)	39 (21.8)	0.794*
Long term care facility	37 (3.0)	0	3 (2.7)	16 (3.4)	8 (2.7)	2 (1.3)	8 (4.5)	0.794*
Transferred to another hospital	9 (0.7)	0	1 (0.9)	5 (1.1)	0	2 (1.3)	1 (0.6)	0.794*
Other	47 (3.8)	1	5	17(0.4)	12	6	6	0.794*
**Mortality**								
Death during hospital stay (%)	45 (3.7)	1 (10)	5 (4.5)	15 (3.2)	12 (4.1)	6 (3.9)	6 (3.4)	0.943 *
30 days mortality all cause (%)	30 (2.5)	0	3 (2.8)	12 (2.6)	8(2.8)	3 (2.0)	4 (2.3)	0.989*
Total mortality	75 (6.1)	1 (10)	8 (7.2)	27 (5.7)	20 (6.8)	9 (5.8)	10 (5.6)	0.948*

*P-value for categorical variables: Chi-square.

**P-value for median: Independent-samples median test.

*Adenovirus excluded from calculations.*

*IE = Infection event; SD = Standard deviation.*

The majority of patients (69%) were discharged to their homes, 23% to short-term care facilities, and 3% to long-term care facilities (Table 4).

We found no significant difference in discharge status of the patients between the different viral RTIs (*p* = 0.794, Chi-square).

#### Mortality

During the hospital stay, 45 patients died (3.7%), ranging from 3.2% in influenza A patients to 4.5% in parainfluenza patients. A further 30 patients died during the 30 days post-discharge (2.5% 30 days all-cause mortality) with no significant difference when comparing the virus types (*p* = 0.943 in-hospital, *p* = 0.989 thirty-days, Chi-square).

Total mortality in the population was 6.1%, with no difference between virus types (*p* = 0.949, Chi-square).

In addition, when using logistic regression, we found no differences in mortality rates when comparing the different virus types (Table 2).

## Discussion

To our knowledge, this study is the first of its kind in Scandinavia, aiming to compare clinical outcome and mortality rates of hospitalized adults with viral RTIs. By investigating clinical outcome in viral RTIs, we discovered that patients suffering from RSV had significantly higher NEWS indicating more severe illness compared with influenza A patients. Comorbidities such as COPD and congestive heart failure were more often found in cases with RSV. The RSV patients also had higher WBC and CRP levels when compared to influenza A, indicating more severe inflammation. These patients were also treated more often with antibiotics before hospitalization (18), during their stay and at discharge. The influenza B patients less often needed respiratory support than the influenza A patients and were rarely prescribed oseltamivir. We found no differences in the need for ICU treatment or mortality when comparing the patients with different viral RTIs.

We found that on admission, patients with RTI caused by RSV, parainfluenza and hMPV were more clinically affected than those with influenza A and influenza B, as judged by the NEWS score ≥5. A perceived more severe disease may be a driver for increased antibiotic prescription in the RSV group. Our finding is in line with the HARTI study, which suggests that hospitalized RSV patients experience a more significant burden of symptoms from the lower respiratory tract, including cough, wheezing, and shortness of breath, during hospitalization and post-discharge (4).

In our study, RSV was more often detected than influenza in patients suffering from COPD. This may be due to protection from previous influenza vaccination, as in Norway, influenza vaccination is recommended and administered free of charge to patients with COPD. The vaccine coverage in the Norwegian population is approximately 30%, but this is thought to be underestimated, due to the fact that many vaccines are administered at care facilities, which do not report to vaccine administration in the national vaccine registry system (19).

The finding is also in line with several studies, which reported that adults admitted to hospital due to RSV more often had underlying chronic lung diseases than patients infected with influenza (10, 12, 20). In addition, patients with multiple comorbidities, e.g., COPD and congestive heart failure, are more likely to develop symptomatic RSV illness (21). In our study, we found that both RSV and parainfluenza patients more frequently had congestive heart failure when compared to patients hospitalized with influenza A. Another study examining hospital admissions for congestive heart failure during the respiratory season discovered that as much as 5.4% could be attributed to RSV infection (22).

Increased viral loads are thought to be associated with an increase in neutrophilic inflammation of the upper and lower respiratory tracts, with the degree of inflammation in part determining the severity of the disease (23). Neutrophilic inflammation in the respiratory mucosa is also believed to predispose to RSV infection (24). In line with this, we observed that RSV patients were overrepresented compared to influenza A in the group with leukocytosis (WBC ≥11.1 × 10^9^/L), with OR 1.8 (1.3–2.6) *p* = 0.001. Higher proportions of leukocytosis among patients with RSV have also been detected in other studies (25, 26). Our results are in line with a study from Spain, where they found elevated WBC and higher CRP levels in RSV infected patients compared to influenza (26).

Patients with community- acquired pneumonia will undergo testing for both viral and bacterial agents immediately after admission and prescribed empirical antibiotics like benzylpenicillin, assessed for clinical improvement daily and treatment adjusted for any microbiological findings according to standard operating procedures at Norwegian hospitals.

Clinicians may be hesitant to discontinue treatment with antibiotics, even when a viral etiology has been confirmed and low values of procalcitonin were observed (27). In a single-center study from Israel, the clinicians were reluctant to discontinue antibiotics despite lack of evidence for a bacterial infection (11). Another study showed that emergency providers only consider RSV as an admission diagnosis in 36% of adults with an influenza-like illness suggesting that RSV is underdiagnosed in adults (28).

It seems that even in cases with known RSV infection, the clinicians are indecisive or halting discontinuation of the antibiotic treatment, when the patient presents with such a high NEWS score, WBC count and CRP level as observed in our study. Also shown in the study from Israel, antibiotic overuse was more common among patients with RSV than those with influenza (77% vs. 69%) (11).

This observation aligns with our results, where hospital records revealed a significantly higher rate of antibiotic treatment in the RSV positives than the influenza A patients. In the earlier mentioned study from Spain, they found that compared to influenza, empirical antimicrobial therapy was more frequently used in patients diagnosed with RSV, and antibiotic withdrawal at the time of diagnosis confirmation happened infrequently (26). Antibiotic overuse in RSV infection has also been demonstrated in several other studies (10, 29).

We found a higher in-hospital mortality rate (3.7%), than 30 days all-cause mortality rate (2.5%), without any apparent difference in mortality when comparing the virus types.

Several studies have also found more or less equal mortality rates when comparing RSV and influenza (22, 30, 31), although some studies reported higher mortality rates in RSV compared to influenza (11, 13).

In our study, we found relatively low in-hospital mortality and 30 days all-cause mortality rates. Falsey et al. found a mortality rate of 8% in hospitalized RSV patients and 7% in hospitalized influenza A patients, and Loubet et al. found in their study an in-hospital mortality of RSV patients of 8%. In a review from 2017, Colosia et al. found a mortality rate of 6–8% in elderly hospitalized RSV patients. When looking at 30 days mortality, Atamna and coworkers reported mortality rates of 5% and 7% for RSV and influenza A, respectively, higher than the 30 days mortality found in our study. Even though our study population is comparable to the patients included in these studies regarding age, the slightly lower mortality rate could be partly explained by the fact that most of our patients were not residents in care homes and less frail.

Our study has some limitations. First, it is a single-center study, and the findings may not be representative of the general Norwegian population. Secondly, the inclusion criteria of PCR-positivity within a period of 14 days may have led to the inclusion of patients with secondary bacterial infections in the study population. Thirdly, the retrospective observational design of this study is limited to the quality of the documentation in the patient’s charts. Lastly, the lack of influenza vaccine status in the patients is considered a limitation of this study.

One of the strengths of our study is the large number of cases included representative of the adult population in the study area with regards to age and gender. Consequently, our results should display the true prevalence of viral RTIs in hospitalized patients from 18 years of age, but probably not be representative for milder non-hospitalized cases. The study was conducted at a large hospital serving the entire population in the Østfold region, ensuring no referral bias regarding special patient groups. Our study included three consecutive winter seasons, allowing us to investigate the morbidity and mortality during an extended period. Therefore, our results are less affected by temporal outcome variations in the circulating viruses. Also, the PCR methods used to screen for the viral RTIs were standardized and identical during these 3 years and regarded as highly sensitive.

## Conclusion

Adult patients hospitalized with RSV infection were significantly more often prescribed an antimicrobial treatment than patients with influenza A. Moreover, a larger proportion of the RSV patients had high WBC, CRP and NEWS scores. These findings highlight the need for further clinical investigations on RSV disease in adults and the elderly. The awareness of long-lasting symptoms after COVID-19 indicate that there is a need for long-term follow up surpassing 30 days, to investigate whether there are semipermanent consequences of common viral respiratory infection in adults.

In addition, identification of the risk factors for more severe RSV disease, especially in the elderly and patients with comorbidities, will be valuable when considering future interventions such as antivirals or vaccines.

## Data Availability Statement

The data that support the findings of this study are available from the corresponding author upon reasonable request.

## Ethics Statement

The studies involving human participants were reviewed and approved by the Regional Committees for Medical and Health Research Ethics (REK ref. 2017/1917 A). The ethics committee waived the requirement of written informed consent for participation.

## Author Contributions

SD, JH, BB, CJ, and SGD contributed to conception and design of the study. SD and BB organized the database. SD, JL, and BB performed the statistical analysis. SD and SGD wrote the first draft of the manuscript. All authors contributed to manuscript revision, read, and approved the submitted version.

## Conflict of Interest

The authors declare that the research was conducted in the absence of any commercial or financial relationships that could be construed as a potential conflict of interest.

## Publisher’s Note

All claims expressed in this article are solely those of the authors and do not necessarily represent those of their affiliated organizations, or those of the publisher, the editors and the reviewers. Any product that may be evaluated in this article, or claim that may be made by its manufacturer, is not guaranteed or endorsed by the publisher.
